# Case of Diffuse Aneurism

**Published:** 1828-01

**Authors:** 

**Affiliations:** the Westminster Hospital.


### ANEURISM.

### *Case of Diffuse Aneurism.* Treated by Mr. Guthrie, at the Westminster Hospital.

Thomas Ellis, æt. seventy-two, admitted December 4th, with a large tumor, situated in the middle of the internal part of the right thigh, into which an opening had been made two days previous to his admission into the hospital, by a medical practitioner. He gave the following account of himself:—About two months since he had been cured of an anasarcous swelling of both inferior extremities, and about the same period he first perceived the swelling of the thigh. It was at first attended with much pain, and that sense of throbbing peculiar to topical inflammation: does not recollect ever to have perceived pulsation, by feeling it with his hand. His medical attendant applied cold lotion, but the pain and swelling still increased. He afterwards applied hot fomentations and poultice, up to the time of the incision. The patient states that the incision made by the surgeon was deep, and that a considerable quantity of fluid and coagulated blood came away; since which he has been losing blood in considerable quantities.

On his admission into the hospital, there was an oozing of arterial blood, for which a tourniquet was applied over the femoral artery, and which restrained it.

At seven P.M. Mr. Guthrie saw him, and immediately stated that, in his opinion, it was a case of diffused aneurism. He was taken to the operating room, and in moving him there was slight arterial hemorrhage. Mr. White and Mr. Guthrie decided that the best chance of saving the man's life was amputation of the limb; to which the patient, after some reluctance, submitted.

The operation was performed by Mr. Guthrie. There was no tourniquet applied. Mr. White compressed the femoral artery with his finger during the operation. There certainly were not four ounces of arterial blood lost. He was removed to bed, and ordered Tinct. Opii gtt. xl.

December 5th.—Has had a tolerable night; feels comfortable; pulse ninety; tongue clean and moist; bowels have not been open.

℞. Potass. Carbon. ʒss.; Aquæ distillat. ℥ss.; Magnes. Sulph. ʒss.; Syr. ʒj. fiat haust. quarta quaque hora sumend. c. Succi Limon. ℥ss.

6th.—Has had a bad night; been delirious; bowels still confined; tongue foul.

To go on with his medicine.

12th.—He has been gradually sinking, and expired this evening.

*Remarks.*—In the morning after the operation, Mr. Guthrie examined the limb, and found the femoral artery not much enlarged, but ruptured in two parts: the continuation of the artery presented no symptoms of disease. Mr. Guthrie observed that this was a case of diffused aneurism, but which had not been attended by the usual diagnostic symptoms; and that a well-informed medical man, (as, from personal knowledge, he could state the practitioner to have been in this instance,) might, for want of the distinguishing symptoms, be mistaken. A large quantity of clotted blood was found in the sac. The artery was ruptured about its entrance into the tricipital canal, but had not been injured by the puncture. The friends would not allow the body to be examined.

